# Delayed care for patients with newly diagnosed cancer due to COVID-19 and estimated impact on cancer mortality in France

**DOI:** 10.1016/j.esmoop.2021.100134

**Published:** 2021-04-17

**Authors:** J.Y. Blay, S. Boucher, B. Le Vu, C. Cropet, S. Chabaud, D. Perol, E. Barranger, M. Campone, T. Conroy, C. Coutant, R. De Crevoisier, A. Debreuve-Theresette, J.P. Delord, P. Fumoleau, J. Gentil, F. Gomez, O. Guerin, A. Jaffré, E. Lartigau, C. Lemoine, M.A. Mahe, F.X. Mahon, H. Mathieu-Daude, Y. Merrouche, F. Penault-Llorca, X. Pivot, J.C. Soria, G. Thomas, P. Vera, T. Vermeulin, P. Viens, M. Ychou, S. Beaupere

**Affiliations:** 1 Centre Leon Berard, Lyon, France; 2 Unicancer, Paris, France; 3 Centre Antoine Lacassagne, Nice, France; 4 Institut de Cancerologie de l’Ouest, Nantes et Angers, France; 5 Institut de Cancerologie de Lorraine, Nancy, France; 6 Centre George Francoise Leclerc, Dijon, France; 7 Centre Eugene Marquis, Rennes, France; 8 Institut Claudius Regaud, IUCT-Oncopole, Toulouse, France; 9 Institut Curie, Paris, France; 10 Institut Bergonié, Bordeaux; 11 Centre Oscar Lambret, Lille, France; 12 Institut Paoli-Calmettes, Marseille, France; 13 Centre François Baclesse, Caen, France; 14 Institut de Cancerologie de Montpellier, Montpellier, France; 15 Institut Jean Godinot, Reims, France; 16 Centre Jean Perrin, Clermont Ferrand, France; 17 Centre Paul Strauss/ICANS, Strasbourg, France; 18 Gustave Roussy, Villejuif, France; 19 Centre Henri Becquerel, Rouen, France

**Keywords:** COVID-19, cancer, delay, diagnosis, treatment

## Abstract

**Background:**

The impact of the first coronavirus disease 2019 (COVID-19) wave on cancer patient management was measured within the nationwide network of the Unicancer comprehensive cancer centers in France.

**Patients and methods:**

The number of patients diagnosed and treated within 17 of the 18 Unicancer centers was collected in 2020 and compared with that during the same periods between 2016 and 2019. Unicancer centers treat close to 20% of cancer patients in France yearly. The reduction in the number of patients attending the Unicancer centers was analyzed per regions and cancer types. The impact of delayed care on cancer-related deaths was calculated based on different hypotheses.

**Results:**

A 6.8% decrease in patients managed within Unicancer in the first 7 months of 2020 versus 2019 was observed. This reduction reached 21% during April and May, and was not compensated in June and July, nor later until November 2020. This reduction was observed only for newly diagnosed patients, while the clinical activity for previously diagnosed patients increased by 4% similar to previous years. The reduction was more pronounced in women, in breast and prostate cancers, and for patients without metastasis. Using an estimated hazard ratio of 1.06 per month of delay in diagnosis and treatment of new patients, we calculated that the delays observed in the 5-month period from March to July 2020 may result in an excess mortality due to cancer of 1000-6000 patients in coming years.

**Conclusions:**

In this study, the delays in cancer patient management were observed only for newly diagnosed patients, more frequently in women, for breast cancer, prostate cancer, and nonmetastatic cancers. These delays may result is an excess risk of cancer-related deaths in the coming years.

## Highlights

• A reduction of the number of cancer patients attending cancer centers was observed in France from January to July 2020.
• This reduction was observed only for newly diagnosed patients.
• The reduction of new diagnosis was more pronounced in women, for breast cancer, and prostate cancer, and nonmetastatic cancer.
• No compensation was observed for the last months of 2020. The magnitude of delays may be larger in centers within the same country.
• An estimation of the excess cancer death resulting from these delays is presented.

## Introduction

The recent coronavirus disease 2019 (COVID-19) epidemic has resulted in a massive mobilization of heath care systems for the management of patients requiring active treatment and intensive care. Resulting delays in the management of other diseases, in particular cancers, have been reported and identified as a potential risk factor for an increased rate of mortality of cancer patients with curable disease in the coming years.^1^, ^2^, ^3^, ^4^, ^5^, ^6^ The magnitude of this increased mortality is not well known.

An extensive number of articles have reported that delays in the diagnosis and management of patients with cancer are associated with an increased risk of death at an advanced stage, an increased risk of relapse, and death in the localized phase.^7^^,^^8^ Reducing the delays in early management is a general strategy proposed to improve patient outcome in low- and middle-income countries,^9^ as illustrated for breast cancer.^10^ Public health strategies of nationwide screening for breast, colorectal, cervix, and lung carcinoma are based on the reduction of the risk of cancer-related deaths with earlier diagnosis.^11^, ^12^, ^13^, ^14^

While delayed diagnosis and cancer treatment increase the risk of death due to cancer, with few exceptions such as indolent lymphomas or low-risk prostate cancers,^15^, ^16^, ^17^, ^18^ the magnitude of the impact of 1-6-month delay in the initial management of curable cancer patients on relapse and death due to cancer remains less clear, and varies considerably across studies for almost all cancer types.^19^, ^20^, ^21^, ^22^ Newly diagnosed cancer patients are particularly at risk of a negative impact of delayed diagnosis and treatment.^19^, ^20^, ^21^, ^22^ In addition, cancer patients are a population at risk of major complications and death due to COVID-19, in all countries, including France.^23^, ^24^, ^25^

France has a national security system based on free health service including national screening programs. Unicancer is the French Federation of comprehensive cancer centers with an exclusive public activity gathering 18 centers treating ∼23% of all cancer patients in this country (unicancer.fr).

We investigated the impact of the COVID-19 pandemic and the first national lockdown in France on the number of patients consulting within Unicancer centers for a new diagnosis of cancer, according to the cancer type.

## Patient and methods

### Unicancer: new patients per center in 2019 and 2020

Unicancer is the French Federation of Comprehensive Cancer centers. Unicancer includes 18 different hospitals in all regions (http://www.unicancer.fr/en/unicancer-group/key-figures). Each Unicancer center was interrogated for the number of total and newly diagnosed patients consulting from 1 January to 31 July of 2019 and 2020, as well as those recorded from 2016 to 2018. Seventeen of the 18 centers were able to contribute to the study. The 18th center did not contribute because it was engaged in a merger precluding a relevant comparison of 2019 and 2020. The total number of cancers treated as well as that of breast, digestive, thoracic, gynecological, head and neck, urological, and hematological malignancies and general characteristics were collected.

### Incidence of cancers in France

We collected information on the overall incidence of cancers in France from the French National Cancer Institute (INCA) website. In 2018, there were 382 000 new diagnoses of solid tumor cancers in France, and 157 400 cancer-related deaths (41%) (https://en.e-cancer.fr/). Raw nationwide data from the publicly available health care system were also used as of 24 November 2020^26^ to extrapolate the reduction in new cases attending Unicancer centers at the national level.

### Analysis of the literature

We analyzed the published literature on PubMed describing the correlation between cancer treatment (surgery, radiotherapy, and chemotherapy) delay and survival. The keywords used for the enquiry on PubMed were ‘delay’, ‘cancer’, ‘diagnostic’, ‘time to treatment’, ‘survival’. This research was conducted in November 2020 and we identified 3840 articles. These articles were further selected with manual screening focusing on delays for treatment initiation in localized phase. Meta-analysis, retrospective reviews, and trials were selected for all cancer types: breast, colorectal, lung, prostate, head and neck, ovarian, uterine, renal cell carcinoma, bladder, lymphoma, and leukemia; a selection is presented on Supplementary Table S1, available at https://doi.org/10.1016/j.esmoop.2021.100134.^21^^,^^27^, ^28^, ^29^, ^30^, ^31^, ^32^, ^33^, ^34^, ^35^, ^36^, ^37^, ^38^, ^39^, ^40^, ^41^, ^42^, ^43^, ^44^, ^45^, ^46^, ^47^, ^48^, ^49^, ^50^, ^51^, ^52^, ^53^, ^54^, ^55^ The level of increase in the risk of cancer death associated with 1 week to 6 months of delay (depending on the studies) was investigated. With the exception of studies in indolent lymphomas and low-risk prostate cancer,^15^, ^16^, ^17^, ^18^ the majority of other studies reported an increased risk of death ranging from 0.5% per week of delay to 169% per 12 weeks of delay depending on cancer types and across studies^21^^,^^27^, ^28^, ^29^, ^30^, ^31^, ^32^, ^33^, ^34^, ^35^, ^36^, ^37^, ^38^, ^39^, ^40^, ^41^, ^42^, ^43^, ^44^, ^45^, ^46^, ^47^, ^48^, ^49^, ^50^, ^51^, ^52^, ^53^, ^54^, ^55^ (Supplementary Table S1, available at https://doi.org/10.1016/j.esmoop.2021.100134).

### Statistics

Comparisons of the different proportions and numbers were conducted using the chi-square test or the nonparametric Mann–Whitney *U* test. In general, the chi-square test was used to compare the number of patients with different characteristics (e.g. sex) in 2020 versus 2019 (for the total first seven months of these 2 years, or for an individual month).

To calculate the potential impact of delays on the risk of cancer-specific deaths, we conducted three different analyses.

1. As a conservative estimate, we selected a hazard ratio (HR) of 1.06 for the increased risk of death related to a 1-month delay in therapeutic intervention as indicated within the recent analysis by Hanna et al.^20^ for frequent cancer types. This HR was applied to all cancer types for simplicity. The studies on other types reported an HR generally superior to this estimate (Supplementary Table S1, available at https://doi.org/10.1016/j.esmoop.2021.100134). The increased risk of death was compared with general death rates in the French population reported in 2018 (157 400 deaths due to cancer and 382 000 new diagnoses of cancer, i.e. 41% death rate; e-cancer.fr). The increased risk of death related to 1 month or multiple months was calculated as follows:
  If the rate of death due to delay is ‘RDdel’; if the rate of death without delay is ‘RD’; HR corresponds to the HR of death related to a 1-month delay, while the HR related to an n-month delay is HR^*n*^. The risk of death due to delay RDdel can be calculated as follows: RDdel = 1–exp [ln(1 − RD) × HR^*n*^]. If *N* is the difference between the number of newly diagnosed patients seen in 2019 minus the number of newly diagnosed patients seen in 2020 per month, the estimated excess number of deaths related to a delay of 1 month (NDexc) is therefore NDexc = *N* (RDdel − RD) for each individual month of the study.
2. Second, we applied the same calculation with two extreme HRs of 1.02 and 1.1 with a similar standard death rate of 41% to describe different hypotheses.
3. Third, we then applied the same calculation described in the first point with (i) specific mortality rates for five individual tumor types [colorectal, head and neck carcinoma, bladder carcinoma, breast carcinoma, and lung carcinoma (as published in the meta-analysis^15^] and (ii) specific mortality rates of these five individual tumors reported in this country in 2018 (e-cancer.fr).

The statistical analyses were conducted with SPSS 23.0 package (IBM, Paris, France).

## Results

### Patients treated and new patients diagnosed in 2019 versus 2020 in Unicancer

**Figure 1:**
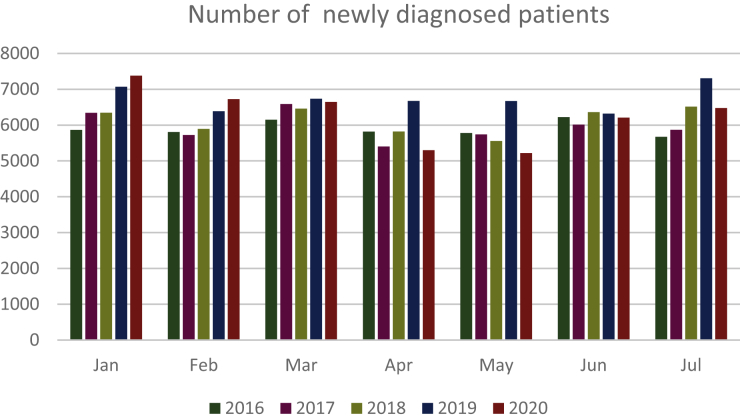
Number of newly diagnosed patients per month from 2016 to 2020.

For the years 2019 and 2020, the total number of cancer patients treated from January to July in Unicancer centers was 90 432 and 89 161, respectively (−1.4%), whereas this number had continuously increased by 2.7%, 3.1%, and 4.6% between 2016 (*N* = 81 666), 2017 (*N* = 83 877), and 2018 (*N* = 86 493) in this network (Figure 1).

The decrease in the number of patients was observed only for patients with a new (2020) diagnosis of cancer (43 947 versus 47 159, −6.8% for 2020 and 2019, respectively), whereas the number of previously (i.e. before 2020) diagnosed patients attending the hospital increased compared with previous years (46 802 versus 44 938, +4.5% for 2020 and 2019, respectively; chi-square *P* < 0.0001). An initial increase in the number of newly diagnosed patients of +4.3% was observed for January 2020 (versus 2019) and +5.3% for February 2020 (versus February 2019), though this was followed by a reduction of −1.4%, −20.6%, −21.8%, −1.8%, and −11.3% from March to July 2020, compared with the same period in 2019 and years 2016-2018 (Figure 1). Over those 5 months, the cumulated decrease reached −11.5% (29 844 versus 33 706) in 2020 compared with 2019 (Table 1).

**Table 1:** Newly diagnosed patients per month per center in high and low COVID-19 incidence zones

Centre	2019 Jan	2019 Feb	2019 Mar	2019 Apr	2019 May	2019 Jun	2019 Jul	2020 Jan	2020 Feb	2020 Mar	2020 Apr	2020 May	2020 Jun	2020 Jul	Total 2019	Total 2020	Ratio 2020/2019
9^a^	676	564	589	581	598	555	622	587	600	625	395	438	455	490	4185	3590	0.86
11^a^	766	750	702	708	764	682	752	785	695	664	520	461	595	686	5124	4406	0.86
13^b^	424	362	329	330	307	321	380	389	312	250	241	255	337	353	2453	2137	0.87
1^b^	316	257	302	314	291	281	310	300	274	278	201	211	265	287	2071	1816	0.88
17^b^	496	435	475	492	488	466	506	500	488	462	335	367	413	440	3358	3005	0.89
5^b^	284	240	279	309	253	241	326	307	256	266	210	218	238	250	1932	1745	0.90
7^a^	513	471	549	534	493	436	574	509	525	482	389	365	494	502	3570	3266	0.91
10^b^	345	321	328	314	334	298	350	344	339	285	279	244	324	346	2290	2161	0.94
12^a^	298	245	283	267	269	304	284	328	274	242	228	259	238	288	1950	1857	0.95
15^b^	438	380	365	382	381	354	415	399	366	415	339	308	392	371	2715	2590	0.95
8^b^	415	356	350	359	352	337	394	399	379	401	298	287	359	345	2563	2468	0.96
3^b^	379	326	392	338	408	340	395	391	368	378	299	320	357	334	2578	2447	0.95
4^a^	224	228	234	247	209	208	243	281	255	246	194	208	208	134	1593	1526	0.96
2^b^	234	253	297	255	260	277	297	310	244	304	179	223	253	280	1873	1793	0.96
16^b^	181	176	198	183	180	176	198	196	194	191	123	127	208	214	1292	1253	0.97
14^b^	613	538	583	583	586	556	642	606	572	609	563	490	551	600	4101	3991	0.97
6^b^	274	281	286	258	285	281	336	294	304	287	291	238	265	278	2001	1957	0.98
Total	7069	6384	6736	6673	6672	6319	7306	7378	6725	6645	5296	5218	6208	6477	47 159	43 947	0.93

COVID-19, coronavirus disease 2019.

Centers were ranked according to the percentage of reduction in new cases in 2020 versus 2019, starting with the greatest reduction.

a Centers in high COVID-19 incidence zones. Centers in high incidence zones did not display a significantly superior reduction in the number of new patients (Mann–Whitney *U* test, *P* = 0.2).

b Cancer centers in other zones.

The median reduction from January to July was 9% (ranging from 13% to 5%) versus 6% reduction (ranging from 13% to 3%) in the five centers in the higher versus lower COVID-19 incidence zone (*U* test, *P* = 0.203).

### Reduced incidence according to stage, gender, and cancer types

**Figure 2:**
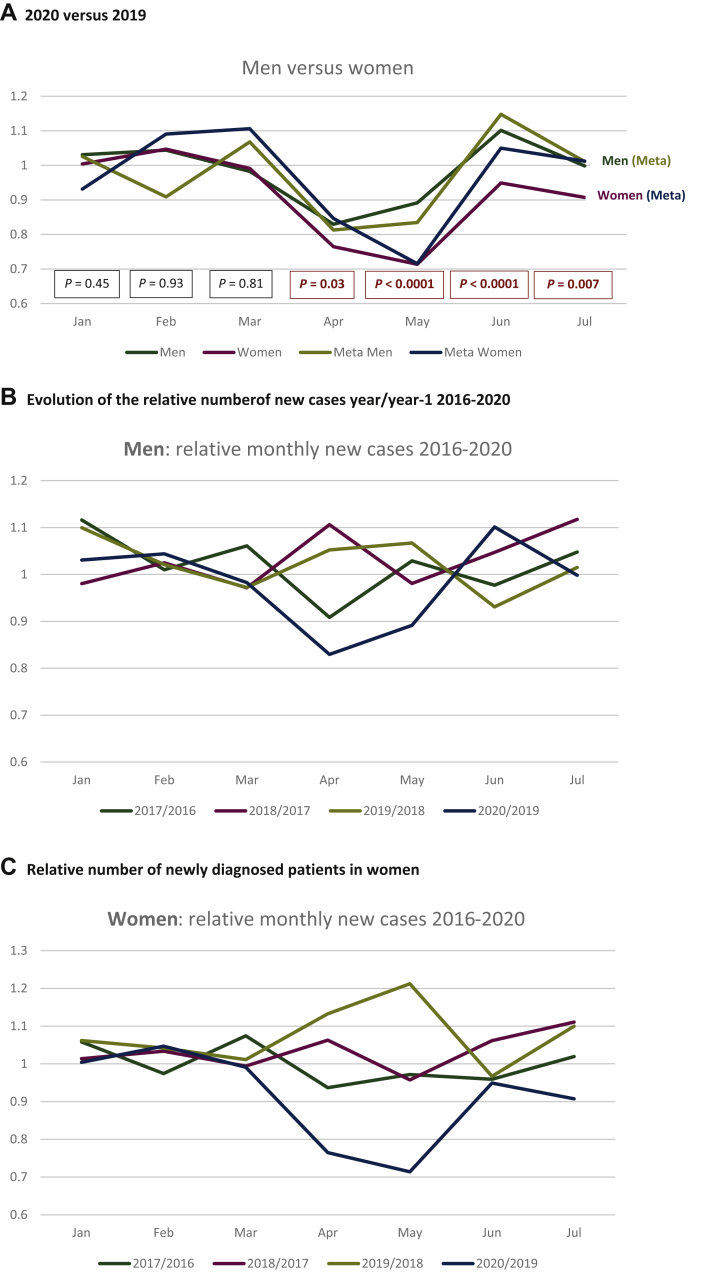
Relative number of newly diagnosed patients in 2020 versus 2019 in men and women. (A) Relative proportion of cases per month in men and women: total numbers (blue and orange curves for men and women, respectively) and with metastases (gray and yellow curves for men and women, respectively). The *P* values correspond to the comparison of the total new cases in men and women per month in 2019 versus 2020. Significant differences were observed from March to July for the total population. The same trend is observed for metastatic patients at diagnosis, but no significant difference was observed. (B) Relative number of newly diagnosed male patients. Relative numbers for each month in 2017 versus 2016 (blue), 2018 versus 2017 (orange), 2019 versus 2018 (gray), and 2020 versus 2019 (yellow). (C) Relative number of newly diagnosed female patients. Relative numbers for each month in 2017 versus 2016 (blue), 2018 versus 2017 (orange), 2019 versus 2018 (gray), and 2020 versus 2019 (yellow).

The reduction of new cancers in women was greater as compared with men, for both patients with and without metastasis (Figure 2A). It was significant from January to July 2020 versus 2019, and specifically in April (chi-square test, *P* = 0.03), May (*P* < 0.0001), June (*P* < 0.0001), and July (*P* = 0.007; Figure 2A). This difference was exclusively observed between 2020 and 2019, in men as well as in women (Figure 2B).

**Figure 3:**
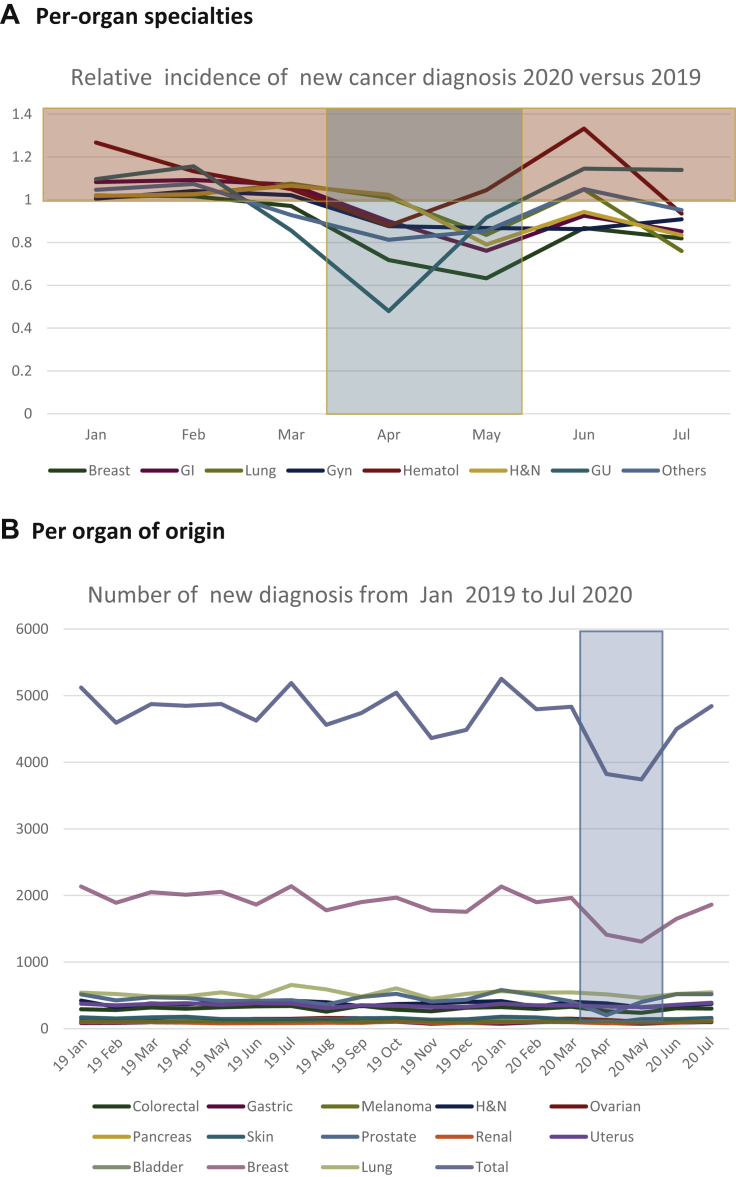

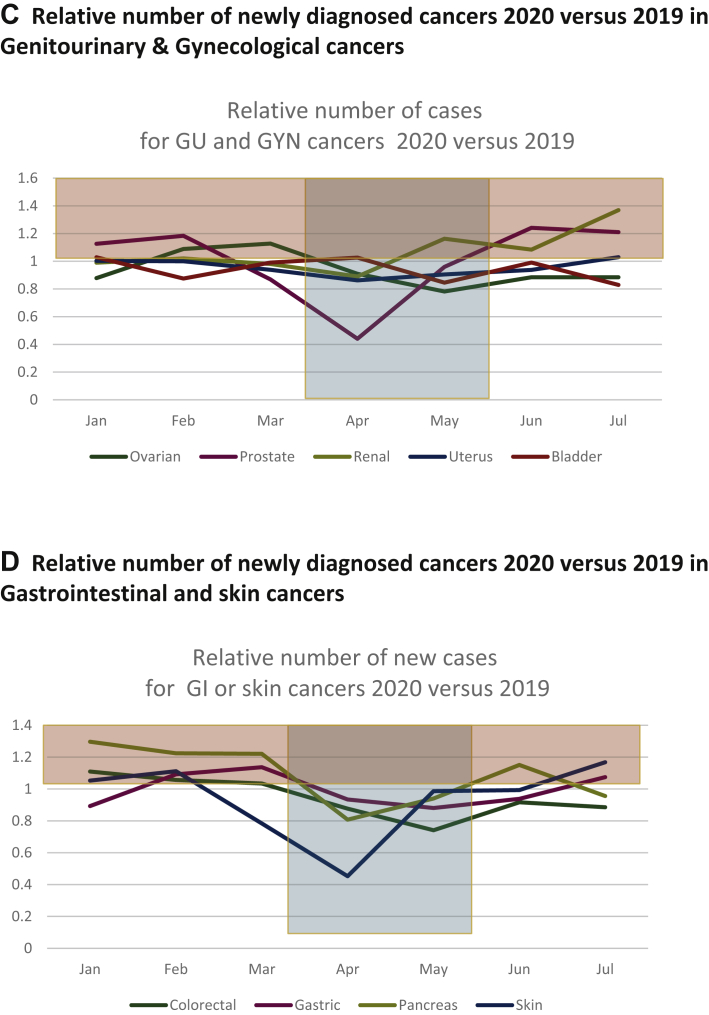
Relative and absolute numbers of newly diagnosed cancers in 2020 versus 2019. (A) Relative number of newly diagnosed patients with cancers of different organs and sites: *x*-axis: months of 2020; *y*-axis: relative proportion of newly diagnosed patients per month in 2020 versus 2019. Highlighted in blue: lockdown 1 period. Highlighted in yellow: higher number of newly diagnosed in patients in 2020 versus 2019. (B) Absolute number of newly diagnosed patients with cancers of different organs and sites: *x*-axis: months of 2020; *y-*axis: total number of newly diagnosed patients per month in 2020 versus 2019, for the different cancer types. Orange curve represents the total. Green curve represents breast cancers. (C) Relative number of newly diagnosed cancers in 2020 versus 2019: genitourinary and gynecological cancers. *x-*axis: months of 2020; *y*-axis: relative proportion of newly diagnosed patients per month in 2020 versus 2019. Highlighted in blue: lockdown 1 period. Highlighted in yellow: higher number of newly diagnosed patients in 2020 versus 2019. (D) Relative number of newly diagnosed cancers in 2020 versus 2019: gastrointestinal and skin cancers. *x-*axis: months of 2020, *y*-axis: relative proportion of newly diagnosed patients per month in 2020 versus 2019. Highlighted in blue: lockdown 1 period. Highlighted in yellow: higher number of new diagnosed in patients in 2020 versus 2019. GI, gastrointestinal; GU, genitourinary; Gyn, gynecological; H&N, head and neck; Hematol, hematological.

We then analyzed the impact on individual cancer types over this 5-month period from March to July 2020. The level of reduction was the highest for breast cancer (8428 versus 10 525, −20.0%), digestive tract cancer (3736 versus 4153, −10.1%), urological cancers (2247 versus 2498, −11.0%), gynecological malignancies (2673 versus 2949, −9.4%), genitourinary malignancies (−10.1%), and head and neck cancers (1889 versus 2038, −7.4%). The impact was lower for lung cancers (2800 versus 2999, −6.7%), and not detectable for hematological malignancies (2133 versus 2058, +3.4%, though −12.2% in April), with a reduction of −8.3% (5714 versus 6227) in the number of new diagnoses for all other cancer types (Figure 3A). Figure 3B shows the number of new cases of the most common cancer types from January 2019 to July 2020 (see also Supplementary Figure S1, available at https://doi.org/10.1016/j.esmoop.2021.100134). The relative reduction of the most frequent urological, gynecological, digestive tract, and skin cancers is presented in Figure 3C and D.

The proportion of reduction was greater for breast cancer versus other gynecological cancers (uterine or ovarian carcinoma) from January to July 2020 versus the same period in 2019 (*P* < 0.001). Similarly, it was larger for prostate cancers versus than other urological malignancies in the same period (*P* < 0.001).

We then assessed the disease stage (metastatic versus nonmetastatic) at diagnosis of newly diagnosed cancer patients from March to July 2020 versus 2019. The relative proportion of metastatic patients was higher in 2020 versus 2019 (24.3% versus 22.2%, *P* < 0.0001).

Within the Unicancer network, this decrease in new diagnoses for the first months of 2020 corresponds to 3862 patients with delayed diagnosis. The extrapolation at the national level [*N* = 31 833 newly diagnosed cancer patients (e-cancer.fr) per months] represents a total of 18 304 cancers with delayed diagnosis in the same period. For breast cancer, the number of undiagnosed patients is 2097 in this network, and 6722 if extrapolated at the national level.

### Estimation of the impact of delayed diagnosis on long-term patient survival

We then estimated the impact of delayed diagnosis and treatment occurring in this period on cancer-specific survival. The impact of the time to initiation of treatment on the survival of specific cancer histotypes and primary sites varies across studies (see Supplementary Table S1, available at https://doi.org/10.1016/j.esmoop.2021.100134).

We first used the HR of 1.06 to estimate the impact of these delays on patient survival in the Unicancer network based on a recent meta-analysis.^20^ This estimation included the reduced number of new patients from March to July 2020 versus 2019, per month, as well as the absence of compensation in the months following the first lockdown, and using a cumulative rate of increased risk of death for patients not consulting for several consecutive months. If we consider a −1.4%, −20.6%, −21.8%, −1.8%, and −11.3% decrease from March to July (Figure 1), respectively, the estimated additional number of deaths per month was 9, 83, 64, 4, and 14 from March to July, with a total of 174 additional cancer-related deaths within Unicancer centers. Nationwide, extrapolation of these estimates for the months of March to July lead to additional deaths per month of 42, 502, 393, 20, and 67 (i.e. an estimated total number of deaths due to delayed diagnosis from March to July 2020 of 1024; Table 2, see footnote a). These tables also include different hypotheses for the additional percentage of patients with delays (on the horizontal bars), and the number of additional months of delays (in the vertical axis, with 1 additional month of delay representing, for instance, all patients diagnosed and treated in September).

**Table 2:** Estimated excess risk of cancer deaths due to delays in diagnosis and treatment in view of the UNICANCER observations of March-July 2020

Additional percentage of patients with delays	0%	−5%	−10%	−15%	−20%	−25%
Calculated number of additional cancer deaths nationwide with a hazard ratio of 1.06 for risk of death per month of delay^15^ for the period from March 2020 to July 2020						
Additional months of delay						
0	1024^a^	1478	1932	2387	2841	3295
1	1381	1992	2604	3215	3827	4438
2	1746	2517	3289	4060	4832	5603
3	2116	3051	3985	4919	5824	6788
4	2492	3592	4692	5791^b^	6891	7990
Calculated number of excess cancer deaths with a hazard ratio of 1.02 for risk of death per month of delay for the period from March 2020 to July 2020						
Additional months of delay						
0	338^a^	488	638	788	938	1088
1	454	654	855	1056	1256	1457
2	570	822	1074	1325	1577	1829
3	688	991	1294	1597	1901	2204
4	806	1161	1516	1872^b^	2227	2582
Calculated number of additional cancer deaths nationwide with a hazard ratio of 1.1 for risk of death per month of delay for the period from March 2020 to July 2020						
Additional months of delay						
0	1715^a^	2476	3237	3998	4759	5520
1	2322	3349	4376	5404	6431	7458
2	2941	4241	5540	6839	8138	9437
3	3570	5145	6719	8294	9868	11443
4	4202	6053	7905	9756^b^	11 608	13459

The number in the left columns indicate the number of additional months of delay in treatment beyond August 2020 in addition to those observed in the Unicancer network: 0: no additional month (i.e. all patients finally treated in August 2020); 1: 1 additional month of delay (i.e. all patients finally treated in September), 2: 2 additional months of delay (i.e. all patients finally treated in October), and so on.

The percentage in the first line represents the additional percentage of patients ‘missing’ in 2020 versus 2019 (i.e. the number of patients observed in 2020 divided by the number of patients diagnosed in 2019) at the nationwide level: 0: similar percentage as for the Unicancer series (i.e. a reduction observed similar to that of the Unicancer series in the same month, nationwide) ; −5%: −5% percentage as compared with the Unicancer series (i.e. −6.4% in March, −25.6% in April), and so on.

The calculated number of additional cancer-specific deaths due to delays with the different assumptions for the hazard ratio is also presented.

a The number calculated with the reduced number of patients observed in the Unicancer series, with the hypothesis that all delayed diagnosis and treatment was completed in August 2020.

b A similar estimation taking into account the observed reduced number of patients with newly diagnosed cancer nationwide in 2020 versus 2019 (data as of 24 November 2020; −23.3%).

These estimates are dependent on three different parameters, namely, (i) the actual percentage of patients with delayed treatment, (ii) the number of months of delay, and (iii) the HR for a 1-month delay.

In a second step, we evaluated the impact of varying this HR. Table 2 shows estimates obtained using an HR of 1.06 (Table 2, first section), a lower HR of 1.02 (Table 2, second section), or a higher HR of 1.1 (Table 2, third section).

On the national social security website,^26^ a reduction of newly diagnosed cancers of 23.3% for the first 7 months of 2020 was described as compared with 2019 (consulted by the authors on the national website 24 November).^26^ This represents a further decrease of 15% in the number of cases diagnosed compared with that measured in the Unicancer centers. Based on this national figure and Unicancer estimates, we then extrapolated nationwide excess mortality due to cancer (Table 2) from 1872 to 9756 excess cancer deaths with the different HRs.

Next, we calculated the number of cancer-specific deaths due to observed delays in Unicancer centers using the specific mortality rate (e-cancer.fr) and the HR of 1.06 per month of delays^20^ for colorectal adenocarcinoma, head and neck carcinoma, bladder carcinoma, and lung carcinoma, and an HR of 1.08 for breast carcinoma. The calculated number of excess cancer-related deaths due to delays in the Unicancer network obtained with the ‘tumor-specific’ method and the ‘one-size-fits-all’ approach was very close (*N* = 120 versus *N* = 132, Table 3). Of note these five tumors accounted for a large proportion of the calculated excess cancer-related deaths aforementioned (132 of the 174).

**Table 3:** Compared number of additional cancer deaths nationwide with a specific hazard ratio and a mortality rate of five tumor types versus the one-size-fits-all approach from March 2020 to July 2020

Parameters	Colorectal	Head and neck	Bladder	Breast	Lung	Total
Number per year in France (2018)	20 120	4298	2448	58 547	15 132	
Number of deaths in France (2018)	7908	1055	1223	12 146	10 356	
Mortality (death/new cases) in 2018	0.39	0.25	0.5	0.21	0.68	
New patients in Unicancer in 2020 (March-July)	1439	1826	514	8194	2590	
New patients in Unicancer in 2019 (March-July)	1626	1977	553	10 115	2701	
‘Missing’ patients in 2020	176	111	36	1921	51	
Hazard ratio per months of delay	1.06	1.06	1.06	1.08	1.06	
Estimated excess deaths with uniform death rate (41%) and uniform hazards ratio of 1.06 per month	9	6	2	108	7	132
Estimated excess deaths with tumor-specific death rate and adapted hazards ratio per tumor	9	6	3	88	14	120

It is important to note that these two estimates are based on the assumptions that from August 2020, all patients with delayed diagnosis would have been managed without further delay. Currently (as of 28 February 2021) the reported number of new patients seen in the Unicancer network in 2020 was 28 900 versus 28 955 from August to November in 2019 (the 2020 figures being 99.8% of that of 2019). No further reduction in new diagnoses was thus observed in this period, but no increased activity was measured that would have compensated the reduced activity during the lockdown.

## Discussion

In this work, we (i) present a description of the reduction of the number of new cases of cancers observed during the first lockdown in France, with an analysis on the type of tumors, sex, and stage, and (ii) propose an estimation of the potential impact on long-term cancer-free survival of patients impacted by these delays.

We describe first the observed reduction in the number of cancer patients seen in the first seven months, including the first lockdown, of the first COVID-19 outbreak in France, from March to July 2020. This was done for 17 of the 18 comprehensive cancer centers in France (Unicancer centers) treating close to one-fourth of all cancer patients in France.

The total number of previously diagnosed cancer patients treated in the Unicancer centers increased in 2020 similar to levels observed in the previous year. Conversely from January to July 2020, there was a reduction of 6.8% of newly diagnosed cancers in this network, versus +4.5% between 2019 and 2018. The reduction was detectable as early as March 2020, while an increase in the first two months of 2020 was recorded in agreement with the trends of the past years. This decrease reached >20% in the 2 months (April and May) of the first lockdown and was observed in July 2020 (−11%). It was not significantly different in centers located in regions with a high incidence of COVID-19 during this period.

The observed reduction was more pronounced in women, for both patients with and without metastases at diagnosis. It was also higher in breast cancers, prostate cancers, and skin cancers. The number of new patients presenting metastases decreased less than those with localized diseases.

These results show that only patients not previously diagnosed with a cancer were those who postponed their consultation to medical services during the first lockdown and in the following months. This was observed for not only tumors with national screening plans (breast, colorectal, cervix cancers), which were interrupted during lockdown, but also for other tumors, indicating that this is not only related to delayed access to screening. The reduced breast cancer screening during this period is likely to have contributed to the level of reduction in new breast cancer diagnosis. The fear of contracting COVID-19 and difficulties in accessing primary care centers or general practitioners are mentioned as possible causes.^1^, ^2^, ^3^, ^4^, ^5^, ^6^

The impact of delayed access to diagnosis and treatment for newly diagnosed patients with cancer on the risk of cancer-related death was the second question explored in this work.

A large number of studies have estimated the increased risk of death due to delays in diagnosis and for the initiation of treatment.^21^^,^^27^, ^28^, ^29^, ^30^, ^31^, ^32^, ^33^, ^34^, ^35^, ^36^, ^37^, ^38^, ^39^, ^40^, ^41^, ^42^, ^43^, ^44^, ^45^, ^46^, ^47^, ^48^, ^49^, ^50^, ^51^, ^52^, ^53^, ^54^, ^55^ These studies are heterogenous in their methodology, and report a variable magnitude of impact across cancer types, depending on the timing of the delay and the nature of the treatment. A selection, by no means exhaustive, of studies is presented in the Reference section and in Supplementary Table S1, available at https://doi.org/10.1016/j.esmoop.2021.100134. Delays, ranging from 7 days to >6 months, for surgery and delays to the administration of chemotherapy or radiotherapy^21^^,^^28^, ^29^, ^30^, ^31^, ^32^, ^33^, ^34^, ^35^, ^36^, ^37^, ^38^, ^39^, ^40^, ^41^, ^42^, ^43^, ^44^, ^45^, ^46^, ^47^, ^48^, ^49^ were all reported as being associated with a reduced cure rate and survival in both localized and advanced phases of a magnitude ranging from 0.5% per weeks of delay up to +169% with 12 weeks of delays in similar cancers.

A recent meta-analysis conducted for breast, colorectal, lung, bladder, and head and neck cancers^20^ showed that a 1-month delay in surgery is associated with an HR of 1.06-1.08 for the risk of cancer-specific death, 1.09 for delay in radiotherapy in head and neck cancer, and variable for adjuvant chemotherapy. In other cancer types, 1-month delays in surgery or neoadjuvant chemotherapy are associated with variable HR often superior in magnitude (summarized in Supplementary Table S1, available at https://doi.org/10.1016/j.esmoop.2021.100134).

Herein, we first selected an HR of 1.06 as a reasonable estimate matching the analysis reported, with the knowledge that lower and higher HRs are reported in various tumor types. The mortality rate for cancer of 41% reported in the national population in France was used for this calculation (e-cancer.fr).

With this uniform HR of 1.06, the number of excess cancer deaths calculated for the first months of 2020 was 1024. This number supposes that no further delays were observed after August 2020, and that the percentage of patients with delays was similar across France. These two hypotheses are, however, unlikely in view of the larger 23.3% reduction in number of new cases observed at the national level on the same period.^26^ For these reasons, a more realistic number of excess cancer death of 5791 is calculated for the first 7 months of 2020. These estimates again suppose that all treatments were initiated shortly after July 2020 which is not known yet. The number of new patients observed between August and November 2020 was stable versus 2019 in Unicancer centers, but no compensation for the backlog of the first 7 months of the year was observed.

Differences in the percentages of patients with delayed diagnosis and treatment, as well as the number of months of delay, and the HR associated with delay all strongly influence the impact of COVID-19 on cancer-specific survival. An HR of 1.02 for a 1-month delay is below that generally reported for most cancer types, whereas a 1-month delay associated with an HR >1.1 is reported in several tumor types (^21^^,^^27^, ^28^, ^29^, ^30^, ^31^, ^32^, ^33^, ^34^, ^35^, ^36^, ^37^, ^38^, ^39^, ^40^, ^41^, ^42^, ^43^, ^44^, ^45^, ^46^, ^47^, ^48^, ^49^, ^50^, ^51^, ^52^, ^53^, ^54^, ^55^ and Supplementary Table S1, available at https://doi.org/10.1016/j.esmoop.2021.100134). Excess cancer deaths calculated with these HRs were intended to provide only a possible estimate of the level of the impact of cancer-related deaths, not an accurate description.

Finally, these estimates do not take in account the yearly increase in new cancer diagnoses, which was +0.9% from 2018 to 2019 on the nationwide social security platform.

The number observed in Unicancer centers thus represents an ‘optimistic’ evaluation of the percentage of patients with delayed diagnosis and treatments which may be greater in nonspecialized centers nationwide.

One of the limitations of this work is to have selected a unique HR for all cancer types. Ideally, a specific HR should be provided for each cancer type and for different stages, histotypes, and ages. The impact of delay per stage and cancer types was reported recently at the national level in the United States,^19^ showing variable HR across different cancer types and stages. In this study, increased time to treatment initiation was associated with poorer survival for stages I and II breast, lung, renal, pancreas, and colorectal cancers, with HRs ranging from 1.005 to 1.030 per week of increased time to treatment initiation.^19^

It is important to note that stage, age, and histotypes are by definition not documented in patients who did not attend to the hospital for diagnosis, and therefore stage-adapted calculations are not possible in this work. The recently reported online tool (OncCOVID) calculates the risk of death due to cancer depending on stage, age, tumor type, and duration of delay to treatment.^56^ While accurate for a single patient, this tool may not be applied to this work for the same reasons, and also because the delays for patient attending the Unicancer centers were outside of our scope. Importantly, the COVID-19-specific cause of death^23^, ^24^, ^25^ is not calculated in this present work which focuses on cancer-specific deaths.

However, we tested cancer-specific HR for five different cancers. Interestingly, the excess mortality calculated with these five tumor-specific parameters was very similar, inferior only by <10% (120 versus 132) to those obtained with the unique mortality rate of 41% and HR of 1.06, as shown in Table 3. Because these five tumors were shown to contribute to 132 of the 174 deaths in excess due to delays, this observation supported the rough estimate of the excess cancer deaths calculated in this work.

Altogether, these results indicate that cancer patients attended hospital consultations for initial diagnosis and treatment with delays in this period, even in comprehensive cancer centers. This was observed for the majority of cancer types, more frequently in women, in breast cancers, and for prostate cancer in particular. It was more frequently observed for nonmetastatic patients. It will result in a significant increase in the number of cancer death in the future for which an estimation is provided in this study. Maintaining access to rapid diagnostic and treatment procedures is an important medical priority in the months to come before the normalization of the health care system expected following the implementation of vaccination.
