# Supplementary material for: Delayed care for patients with newly diagnosed cancer due to COVID-19 and estimated impact on cancer mortality in France
Source: ESMO Open. 2021 Apr 17;6(3):100134. doi: 10.1016/j.esmoop.2021.100134 (PMC8134718; doi:10.1016/j.esmoop.2021.100134)
Supplement: Supplementary Table S1 and Figure S1 [file mmc1.docx]

**Supplementary Table 1: A selection of studies exploring the impact of time to treatment initiation of overall survival**

TTI: time to treatment initiation; RT: radiotherapy; CT: chemotherapy; NR: not reported; PORT : post-operative radiotherapy; S: statistically significant; NS: non significant statistically. Highlighted in red: significant detrimental impact of delay on OS; Highlighted in green: significant protective impact of delay on OS;

**Supplementary Figure 1 : Absolute number of newly diagnosed patients with cancers of different organs and sites:**

Suppl. Figure 1A: Cancer types with a monthly incidence from 200 to 700

***
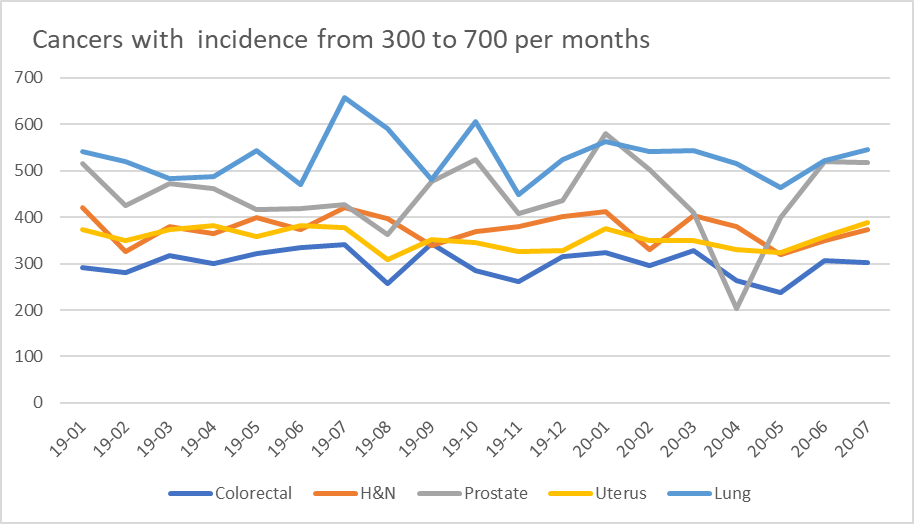
***

Suppl. Figure 1B: Cancer types with a monthly incidence from 100 to 200

***X axis:*** months of 2020, ***Y axis:*** Total number of newly diagnosed patients per months in 2020 vs 2019, for the different cancer types.
